# Anaerobes and Bacterial Vaginosis in Pregnancy: Virulence Factors Contributing to Vaginal Colonisation

**DOI:** 10.3390/ijerph110706979

**Published:** 2014-07-10

**Authors:** Charlene W. J. Africa, Janske Nel, Megan Stemmet

**Affiliations:** Department of Medical Biosciences, University of the Western Cape, Private Bag X17, Bellville 7535, Cape Town, South Africa; E-Mails: 2917239@uwc.ac.za (J.N.); megcstem@gmail.com (M.S.)

**Keywords:** pregnancy outcomes, bacterial vaginosis, female morbidity, microbial ecology

## Abstract

The aetiology and pathogenesis of bacterial vaginosis (BV) is unclear but it appears to be associated with factors that disrupt the normal acidity of the vagina thus altering the equilibrium between the normal vaginal microbiota. BV has serious implications for female morbidity, including reports of pelvic inflammatory disease, adverse pregnancy outcomes, increased susceptibility to sexually transmitted infections and infertility. This paper reviewed new available information regarding possible factors contributing to the establishment of the BV vaginal biofilm, examined the proposed role of anaerobic microbial species recently detected by new culture-independent methods and discusses developments related to the effects of BV on human pregnancy. The literature search included Pubmed (NLM), LISTA (EBSCO), and Web of Science. Because of the complexity and diversity of population groups, diagnosis and methodology used, no meta-analysis was performed. Several anaerobic microbial species previously missed in the laboratory diagnosis of BV have been revealed while taking cognisance of newly proposed theories of infection, thereby improving our understanding and knowledge of the complex aetiology and pathogenesis of BV and its perceived role in adverse pregnancy outcomes.

## 1. Introduction

Preterm birth (PTB) is the leading cause of neonatal morbidity and mortality world-wide, yet, the underlying causes remain largely unknown, with increased incidence reported due to intrauterine infections [1,2]. Bacterial vaginosis (BV) is a common reproductive tract infection amongst women of reproductive age [3] and has been implicated as a risk factor for adverse pregnancy outcomes such as preterm birth, recurrent abortions, post-abortal sepsis, early miscarriages and still births [2,4,5,6,7]. Infections leading to preterm birth and other complications of pregnancy may extend beyond delivery and create serious and sometimes life-threatening consequences for the neonate [8]. However, many infections are subclinical [9] and so our knowledge of the role played by BV in PTB remains limited and inconclusive.

Previously considered to be an infection initiated by the overgrowth of *Gardnerella vaginalis* with a concomitant loss of H_2_O_2_-producing lactobacilli, new evidence points to BV as being a polymicrobial condition, involving synergistic mechanisms between vaginal colonisers, many of which are anaerobic bacteria. Confirmation of this theory is found in the resistance to metronidazole of many BV infections, and the finding that the volatile fatty acids responsible for the “whiff test” are products of the metabolic activity of a host of anaerobes other than *G. vaginalis* [10]. Although not classified as a sexually transmitted infection (STI) *per se*, the risk for sexual transmission of certain BV-associated bacteria has been reported to increase in lesbian and heterosexual women with multiple sexual partners [11,12,13].

## 2. Methodology

Citation databases included Sciencedirect, PubMed, LISTA (EBSCO), and Web of Science, using the terms “bacterial vaginosis”, “vaginitis”, “vaginal discharge”, “aetiology of vaginosis”, “anaerobes in vaginosis”, “preterm birth and vaginosis”, “preterm delivery and asymptomatic infections”, “pathogenesis of bacterial vaginosis”, “virulence factors of vaginal anaerobes”, “diagnosis of vaginosis” and “vaginal microbial ecology”. All titles and available abstracts were screened and where relevant, full texts retrieved. In addition, reference lists of retrieved papers were examined for similar papers which could be relevant. More than 700 papers were retrieved in the initial search but only 161 papers pertaining to specific BV-associated anaerobes in pregnancy and published between the years 2000 and 2014 were included, except for a few specific earlier papers on which diagnostic methods were based. Data regarding bacteria detected, method of detection, type of study (case/control or cohort) virulence mechanisms and implication in adverse pregnancy outcomes were collected and summarised. While not claiming to be all-inclusive, an attempt was made to include the viewpoints and research outcomes which contributed new and pertinent data. Overt differences in diagnosis, proposed aetiology, sampling, detection methods, population groups and periods of gestation complicated a meta-analysis.

## 3. Results and Discussion

### 3.1. Transition from the Lactobacillus-Associated to the BV-Associated Vaginal Biofilm

The vaginal ecosystem is established over a number of years. The dynamic environment of the vagina is influenced by factors such as hormonal fluctuations, menstruation, douching, hygiene, pregnancy, breastfeeding and sexual practices [14,15,16,17,18,19,20,21,22,23,24,25,26,27]. Skin commensals and microbiota normally inhabiting the bowel are the original colonisers of the young, healthy female vagina. Aerobic lactobacilli persist in the vagina for weeks after birth while the vaginal pH remains acidic. The pH of the vagina converts to neutral during early childhood, remaining so until puberty. A plethora of different microbial species co-exist in this vaginal ecosystem, 70%–90% of which are lactobacilli. During puberty and menarche, dramatic hormonal and physical changes occur in the vaginal environment favouring the colonisation of lactobacilli [26]. Although identified in both BV-positive and BV-negative women, H_2_O producing lactobacilli numbers are reported to be significantly increased in BV-negative females [28]. Their population dominance is considered to be beneficial in maintaining the health of the female, through their production of hydroxyl radicals, lactic acid, bacteriocins, hydrogen peroxide and probiotics [5]. After menopause, or when the vaginal lactobacilli are depressed or removed, hydrogen peroxide is no longer produced and the pH of the environment increases, thus facilitating the establishment of BV-associated microbial communities in the vaginal biofilm [23,29]. However, not all healthy females have vaginal microbiota dominated by lactobacilli [5], and other lactic acid producers such as *Atopobium vaginae, Leptotrichia* and *Megasphaera* have also been suggested to assist in maintaining the acidity of the vagina, [30,31]. With BV considered a non-specific (predominantly anaerobic) polymicrobial infection and the identities of many of the vaginal microflora remaining elusive [32], determining the health of the vaginal ecosystem appears to be subjective.

Two species of lactobacilli have frequently been found to colonise the healthy vagina, namely, *Lactobacillus gasseri and Lactobacillus crispatus* both members of the *Lactobacillus acidophilus* complex [33,34]. While *L. crispatus* and *L. jensenii* have a better ability to adhere to vaginal cells [35], *in vitro* studies have reported that acid and hydrogen peroxide production by *L. acidophilus* and *L. casei* are able to inhibit the growth of BV-associated microbes such as *Gardnerella vaginalis*, *Mobiluncus*, *Bacteroides* and species of anaerobic cocci, while decreased production of bacteriocins and hydrogen-peroxide by lactobacilli enhances the growth of *G. vaginalis*, *Prevotella bivia*, *Mobiluncus*, *Peptococcus* species and *Peptostreptococcus anaerobius* by the production of ammonia, acetic, succinic acids and amino acids respectively [36].

*Lactobacillus iners* has been recommended as a marker of the imbalance of the vaginal microflora leading to BV [33,37,38] and its presence correlated with colonisation of other BV-associated bacteria such as *Megasphaera*, *Leptotrichia* and *Eggerthella* [39]. Thus the significance of the relative ratio of *Lactobacillus iners*, *Atopobium vaginae* and other anaerobes in BV provides substantial information for its diagnosis [38]. *L. gassei* and *L. crispatus*, have been associated with health, while a negative correlation has been reported between *L. gasseri* and *L. iners*; *L. gasseri* and *Atopobium*, *Prevotella* and *G. vaginalis* all of which are associated with BV [37,39,40,41]. *L. iners* is dominant after treatment for BV, and has been reported together with *L. crispatus* in women without BV [34] suggesting that *L. iners* may be an opportunistic pathogen whose prevalence and pathogenic potential is enhanced by *L. gassei*, an assumed later coloniser than *L. crispatus*.

Several mechanisms have been proposed for the establishment of the BV biofilm, namely, stress, sexual practices, microbial synergism (one organism inducing the ideal growing environment for another) or antagonism (inhibition or killing of one organism by another). Because of the polymicrobial nature of infection, growing resistance to metronidazole and other antimicrobial treatments have been reported [42]. The outcome of any infection is usually determined by the initial response of the host to invasion by an infective agent, followed by chemotaxis (the migration of polymorphonuclear neutrophils (PMNs) towards the locus of infection). This initial response to the recognition of chemotactic factors results in phagocytosis. In an effort to fully understand BV, clinicians made a distinction between vaginitis and vaginosis by defining bacterial vaginitis as an infection of the vagina associated with inflammation of the vulva, while vaginosis was defined as the degradation of the normal flora of the vagina in the absence of an associated inflammatory response [43]. The inhibition of PMN chemotaxis by virulence factors expressed by certain of the BV-associated organisms, may explain the absence of inflammation.

In order to be implicated in the aetiology of BV, a specific bacterium should demonstrate an increase in BV and be reduced or eliminated in the healthy vagina. BV has been attributed to an imbalance of the normal vaginal microbiota with many of the anaerobic bacteria detected in BV showing great pathogenic potential either individually or as a consortium. Symbiotic relationships appear to play an important role in the pathogenesis of BV, thereby implicating several species in its aetiology. They are examined below in the light of their effect on host defences, provision of essential nutrients for growth and survival, alteration of the environment and expression of virulence factors.

### 3.2. Anaerobes Implicated in the Aetiology of BV

*Gardnerella vaginalis* has for a long time been seen as one of the most important candidate bacteria in BV [27,44,45,46], with its numbers being inversely proportionate to lactobacilli in health and disease [17,18]. However, recognising only *Gardnerella vaginalis* as the overt pathogen in BV would be inaccurate, especially as *G. vaginalis* has shown a high sensitivity (100%) but low specificity (49%) for BV by also being detected in the absence of BV [14,16,28,47]. Synergy between *G. vaginalis* and *Atopobium vaginae* has been proposed as they are often detected together in BV [32,48,49,50,51,52] and only rarely has *A. vaginae* been detected in the absence of *G. vaginalis* [53] or in health [54,55]. *A. vaginae* was first implicated in BV in 2004, since then several studies have emerged associating this anaerobe with >80% of BV cases [54,56,57,58,59,60,61]. Furthermore, a specific consortium including *A. vaginae,* and *Leptotrichia* was found to enhance the pathogenic potential of *Megasphaera* in BV [62], suggesting a role for *A. vaginae* in the aetiology of BV. *Megasphaera* has a symbiotic existence in the vaginal biofilm [63] and appears to be sexually transmitted, being strongly associated with women who have sex with women and with heterosexual women reporting an increased number of sexual partners [15,25].

Taxonomically unidentified bacteria designated BVAB (bacterial vaginosis associated bacteria) have morphologic features distinctly different from *Atopobium*, *Mobiluncus*, or *Gardnerella* and have been observed microscopically in patients with BV, attached to vaginal epithelial cells typical of the clue cells that characterise BV [47,64]. BVAB have been reported in HIV-positive women with BV [65] and are considered to be acquired from external reservoirs such as the anus [66]. Also associated with BV infection in HIV-positive women are *M. hominis* and a combination of *Bacteroides*, *Prevotella*, *Leptotrichia*, *Atopobium* and *Gardnerella* [49,67,68,69,70,71,72] known to attract CD4 cells to the mucosa, with a concomitant reduction in lactobacilli [67]. Among the Gram-positive anaerobic cocci known to colonise the vagina are *Peptococcus*, *Peptostreptococcus*, *Peptoniphilus* and *Anaerococcus*. However, Gram-positive anaerobic cocci have not received the attention they deserve in studies of the vaginal biofilm despite their perceived pathogenic potential and their production of metabolic end products which favour the growth of other BV-associated microbes such as *Megasphaera* [63]. Furthermore, an association between endometritis and BV-associated anaerobic Gram-positive cocci has been established [73], confirming their role in reproductive morbidity.

*Leptotrichia amnionii* and *Sneathia sanguinegens* (previously *Leptotrichia sanguinegens*) are known colonisers of the oral cavity and like other oral anaerobes implicated in the aetiology of periodontal disease such as *Tannerella forsythia*, *Treponema denticola*, *Prevotella intermedia*, *Aggrigatibacter actinomycetemcomitans* (Aa) and *Porphyromonas gingivalis*, have also been associated with BV [74]. Their presence in the vagina may be due to translocation during oral-gential sexual practices since they are rarely found in sexually unexposed women [15]. Known to co-colonise with *G. vaginalis* and other anaerobes such as *Prevotella* in cases of BV [49,58], *L. amnionii* and *S. sanguinegens* although significantly increased in women with cervical cancer, show no correlation with human papillomavirus which has been implicated in the aetiology of cervical cancer and their detection in healthy women suggests that their pathogenicity may be opportunistic [75].

### 3.3. Diagnosis of Bacterial Vaginosis

Diagnosis of BV includes clinical examination and microscopy [76,77], using Amsel criteria and Nugent scoring [78,79]. BV is characterised by a discharge (often white or yellow) with a fishy odour following the addition of 10% potassium hydroxide to the vaginal fluid, vaginal pH > 4.5 and the microscopic scoring of bacterial morphotypes along with the presence of “clue cells”, vaginal epithelial cells with boarders coated with bacteria [80]. It may also be asymptomatic [9,26] which explains why it is often misdiagnosed [30]. It may be differentiated from aerobic vaginitis, which is characterised by the absence of succinate, increased sialidase activity and a host response resulting in the increased production of cytokines such as interleukin-1, -6 and -8 [43,81].

Laboratory-based diagnostic methods for BV include culture and anaerobic metabolic activity analysis such as the assessment of the production of short-chain volatile and non-volatile fatty acids as end products of anaerobic metabolism, and the production of specific enzymes, [82,83,84,85,86]. Detection of fatty acids in vaginal fluid [87,88,89] can differentiate between health (major lactic acid) and BV (major succinic and acetic acids) as well as monitor the effects of treatment for BV. Culture-based and molecular approaches have identified several anaerobic species in BV [18,28,39,40,48,53,90,91] with molecular methods having overcome many of the problems associated with culture and revealing species not previously reported. This has indeed awakened our awareness of the complexity of the microbial biofilm of BV (Table 1) as well as the risk and treatment of ascending subclinical infections [92]. Of the many bacteria implicated in the aetiology of BV, *Leptotrichia amnionii* and *Eggerthella* species are the only two species found to correlate with all of the Amsel criteria used for the diagnosis of BV [34] and in cases where no discharge is present, improved grading of bacterial morphotypes in a wet mount or Gram stain of vaginal fluid has been recommended as an alternative to Amsel criteria for the diagnosis of BV [93].

**Table 1:** Anaerobic microbial consortia most frequently associated with bacterial vaginosis and preterm birth.

Reference	Gram-negative Anaerobes					Gram-positive Anaerobes									
	La	Ss	Por	Pr	Di	Av	Gv	Mob	Li	BVAB	Egg	Meg	Anaer	Ps	Pep
[9]	x			x	x	x	x				x				
[10]	x	x				x					x	x			
[15]						x	x			x		x			
[18]				x		x	x								
[24]	x	x		x		x	x				x				
[25]	x	x				x	x			x		x			x
[34]	x	x				x	x			x	x				
[50]			x	x		x			x			x	x	x	x
[55]							x	x	x		x	x		x	x
[58]	x	x	x		x	x	x		x		x	x	x	x	x
[64]	x	x				x				x					
[94]	x			x	x	x	x	x	x		x	x		x	x
[95]	x	x		x		x	x				x	x			
[96]				x			x	x							
[97]	x			x								x			
[98]	x	x				x				x					
[99]		x				x	x								
[100]	x					x						x			

Notes: Av = *Atopobium vaginae*; Mob = *Mobiluncus*; Li = *Lactobacillus iners*; BVAB = BV-associated bacteria (unidentified); Egg = *Eggerthella*; Meg = *Megasphaera*; Anaer = *Anaerococcus* (previously *Peptostreptococcus*); Pep = *Peptoniphilus* (previously *Peptostreptococ*).

Since the focus of this paper is directed at the microbes implicated in the aetiology and pathogenesis of BV and the role it may play in PTB, it will not elaborate on the details of these different diagnostic approaches, the strengths and weaknesses of which have previously been summarised [101].

### 3.4. The Pathogenic Potential of BV-associated Anaerobes

#### 3.4.1. Co-Aggregation and Attachment

*G. vaginalis* has been reported to possess innate pathogenic potential [44] with two genomically different forms described: one being a commensal form slightly adhesive to epithelial cells, and the other, a pathogenic form found to be strongly adhesive to epithelial cells [33]. Because adherence is a vitally important virulence factor in the pathogenesis of BV, the ability of *G. vaginalis* to adhere to vaginal epithelial cells may well pave the way for other microbes such as *Peptoniphilus* and *A. vaginae* to become established in the BV-associated vaginal biofilm by co-aggregating with *G. vaginalis* in order to establish infection [44,102].

Other species such as *Fusobacterium* which also do not adhere well to vaginal epithelial cells [44], are able to facilitate co-aggregation between species such as *Prevotella* [103] and *Bifidobacterium* [104] in polymicrobial infections [105,106] by acting as a bridge between early and late colonisers in biofilm formation [107,108,109] and by supporting the growth of obligate anaerobes such as *Porphyromonas*, [110,111] in aerated and carbon dioxide depleted environments [110,111,112]. By adhering to other vaginal microbes [113], *F. nucleatum* is able to mask the surface components that are recognized by H_2_O_2_-producing lactobacilli and prevent detection by antagonistic microflora thus allowing for its integration into the developing vaginal microbial community associated with BV [114]. *Bifidobacterium* and *Ureaplasma urealyticum* have the ability to attach to erythrocytes and other eucaryotic cells, [115] and have therefore been reported to be independently associated with BV [9]. However, *Bifidobacterium adolescentis*, *Bifidobacterium longum*, *Bifidobacterium breves*, *Bifidobacterium bifidum* and *Bifidobacterium catenulatum* have been detected perianally only and not in the vagina [116], possibly due to weaker adherence mechanisms. While unable to co-aggregate directly with *Actinomyces naeslundi* or with *Veillonella parvula*, *Bifidobacterium* is known to attach to biofilms of *A. naeslundii* and *V. parvula*, in the presence of *F. nucleatum* [104] and may further contribute to the formation of the vaginal biofilm by producing amino acids which provide energy sources to other anaerobic bacteria such as *Eggerthella lenta* (previously *Eubacterium lenta*).

#### 3.4.2. Production of Volatile and Non-Volatile Fatty Acids

Volatile fatty acids such as isovaleric or caproic acid are produced by species of Gram-positive cocci which induce alterations of the shape, composition and growth of eukaryotic cells [88] and may thus threaten pregnancy. Non-volatile fatty acids produced by vaginal anaerobes have been shown to inhibit chemotaxis and other PMN functions [88,117]. Other fatty acids produced by vaginal anaerobes include formic acid produced by *Atopobium* and *Eggerthella*, propionic acid produced by *Propionibacterium*, lactic acid produced by *Bifidobacterium*, *Atopobium*, *Actinomyces*, *Collinsella aerofaciens* (previously *Eubacterium aerofaciens*), *Lactobacillus*, *Leptotrichia amnionii* and *Sneathia sanguinegens*, butyric acid produced by *Eubacterium*, *Slackia*, *Gram-positive cocci* and acetic and succinic acids produced by anaerobic Gram-positive cocci, *Mobiluncus*, *Prevotella*, *Atopobium*, *Bifidobacterium*, *Eggerthella*, *Actinomyces* [118]. The production of malic acid and trimethylamine by *Mobiluncus* have been associated with vaginal irritation and the vaginal odour associated with BV, while *Fusobacterium nucleatum* is known to produce large amounts of ammonia, butyrate and hydrogen sulfide and thus, along with supporting the growth of other anaerobes, could also be contributing to the malodour associated with BV [119,120].

The production of amines such as putrescine and cadaverine by anaerobic bacteria, resulting in an increase in pH and favouring the growth of other anaerobes implicated in BV is an example of bacterial synergy in the establishment of the vaginosis biofilm.

#### 3.4.3. Enzyme and Lysine Production

Mucin degrading enzymes produced by BV-associated bacteria deplete protective host mucous barriers [121] and facilitate adhesion to and colonisation of the underlying epithelium [122]. The production of collagenase and fibrinolysins by *Prevotella bivia*, *Prevotella disiens* and *Leptorichia* are reported to play a role in pathogenesis by degrading mucosal protective factors and detachment of vaginal epithelial cells [82] thereby contributing to the production of a vaginal discharge. Cytolysins are produced by *L. iners* [123] and *G. vaginalis*, causing cell death by activating the protein kinase pathway in human epithelial cells [44,124]. The production of IgA antibodies to vaginolysin (cytolysin produced by *G. vaginalis*) as a result of the mucosal immune response, further supports the role of vaginolysin in the pathogenesis of BV [125]. *G. vaginalis* also produces haemolysin, liberating nutrients for the feeding of other anaerobic bacteria. It has been suggested that *U. urealyticum* is the most virulent of the mollicutes [115], expressing haemolytic activity and secretion of enzymes such as elastase, IgA protease (reducing mucosal immunity), phospholipase C, [126], and urease which hydrolyses urea to cytotoxic ammonia. Vaginal sloughing is facilitated by the production of sialidase, prolidase [117,121] and putrescine by *G. vaginalis*, *Mobilincus* [122] *Megasphaera*, [62], *Bactroides fragilis* [122] and *Prevotella bivia* [82,117]. Proteolytic enzymes produced by many anaerobic species [44] damage oviduct mucosal surfaces, render the cilia unable to beat, and could possibly lead to infertility [127,128].

### 3.5. Bacterial Vaginosis and Its Threat to Pregnancy

Vaginal mucosal cells and their glycoproteins are major components of the barrier which protects against infection. Cervical mucous protects the upper reproductive tract from microbial invasion. Among the antibacterial factors contained in mucous are lactoferrin, lysozyme and secretory IgA. The amount and viscosity of mucosal flow is determined by the cervical mucins and thus reduced viscosity of the mucous allows for better infiltration of pathogens into the reproductive tract [122]. Evidence is mounting that ascending subclinical intrauterine infections in the gravid female may threaten pregnancy due to a host of different proinflammatory endogenous vaginal colonisers. By promoting colonisation of the upper reproductive tract, mucin degrading enzymes produced by BV-associated bacteria sometimes cause chorioamnionitis and other adverse pregnancy outcomes. Chemotactic cytokines are produced by several cell types, including injured tissues, and by acting on leucocytes, are involved in stimulating inflammatory mediators [26]. High vaginal bacterial loads may cause an increase in cytokine levels [129] particularly when *G. vaginalis*, *F. nucleatum*, staphylococci and streptococci are present [92]. *G. vaginalis* and *A. vaginae* frequently occur together in PTB [130,131], with *A. vaginae* also being detected in endometritis [132] a condition which may occur not only amongst non-pregnant women (in pelvic inflammatory disease), but could also occur after child birth or as a result of Casaerian delivery. Other BV-associated microbes associated with PTB include *Mobiluncus*, *Mycoplasma* [133] along with *Bacteroides ureolyticus* [134,135] and *Fusobacterium* [136].

*L. amnionii* and *S. sanguinegens* have been reported in pregnancy [137], and ascending infections involving the uterus, foetal membranes and fallopian tubes [138]. Being increasingly reported from obstetrics cases [85] they are associated with spontaneous abortion [138], septic abortion [139], PTB [86,140], peripartum fever [141] postpartum infection [138,141], and bacteraemia [142].

Although considered to be an indicator organism for BV [143,144], the role of *Megasphaera* in preterm birth remains speculative. *M. hominis* appears to increase the prevalence of *U. urealyticum*, *C. trachomatis* [145], BV and preterm delivery [146]. Together, *M. hominis* and *U. urealyticum* have been reported in cervicitis, pelvic inflammatory disease and obstetrical pathologies including premature delivery, premature rupture of membranes, chorio-amniotitis and pregnancy loss [9,145,147,148], while *U. parvum* has been associated with preterm birth and late spontaneous abortion [149]. The role of *M. genitalium* in both BV and pregnancy outcomes is less well defined since its presence appears to be dependent on other vaginal colonisers. The production of sialidase and other proteolytic enzymes capable of damaging host tissues and threatening pregnancy may implicate many of the anaerobic vaginal microflora in adverse pregnancy outcomes [121,150].

## 4. Summary and Conclusions

Anaerobes dominate the microbiota of the human skin and mucous membranes and are thus commonly implicated in bacterial endogenous infections. Their identification and treatment are complicated by their inability to grow in routine culture thus resulting in their presence being missed or overlooked. The use of 16S rRNA gene sequence-based analyses have frequently been used for the study of the vaginal microbiota, thus revealing the presence of many anaerobic species not previously detected by culture [57,58]. Our knowledge of the diversity of species associated with BV has thus increased as detection methods have improved [44,94].

The polymicrobial ecosystem of the vagina complicates the determination of the microbial aetiology of BV. Using culture-independent techniques, attempts have been made to differentiate the diversity of vaginal microbiota in BV-positive and BV-negative women [62,95,151], but there is no consensus regarding the microbial communities which bring about the change from a commensal microflora to an opportunistic microflora causing BV. Many of the studies reported, differ in patient selection, population groups, sampling techniques, diagnostic methods and laboratory detection methods with some studies (particularly molecular studies) selecting for the presence of specific species only and others (cultural studies) limited by the lack of nutrients required for the growth of fastidious species, thus leading to either over or under-representation of the prevalence of particular species.

The HIV-exposed foetus is at risk for PTB [152] thus the viricidal effect of H_2_O_2_-producing lactobacilli and the stimulation of growth factors by BV-associated bacteria have a direct impact on foetal and neonatal morbidity and mortality due to maternal HIV status [153]. However, conclusive evidence that treatment of BV reduces PTB is lacking thus BV is not considered by many as being a specific marker for PTB and focus is thus directed to the development of other biomarkers for adverse pregnancy outcomes. It has been suggested that aerobic vaginitis is a more serious threat for adverse pregnancy outcomes than BV [43] since it appears to be more damaging to the healthy vaginal environment than BV [81] and the increased cytokine production is thought to increase the risk of preterm delivery, chorioamnionitis and funisitis of the foetus [154].

Whether or not screening for BV, prevents PTB remains to be debated [155,156,157]. While some recommend that treatment of BV reduces the risk of PTB, others report an increase in PTB following treatment for BV [158]. Meanwhile, BVAB3 an anerobe prevalent in BV, was found to decrease the risk for PTB [133], a finding which may explain why come cases of BV may be involved in PTB and others not. Inconsistencies in clinical and laboratory detection methods used for the monitoring of treatment have a direct impact on success rates [76,83,155,159] thus complicating a comparison between the presence/absence of BV in PTB and full-term birth (FTB) [156]. Because different bacteria have different associations with Amsel diagnostic criteria, this may explain discrepancies often reported between Amsel criteria and microscopy [34]. Amsel clinical criteria provide no information on the vaginal microflora and while microscopy may provide information pertaining to the morphology of the vaginal bacteria, it provides no confirmation of the species present. Although strongly associated with BV, many bacteria have not been taxonomically identified [47,58], further adding to the complexity of the matter.

With some species associated with both health and disease, one needs to examine their pathogenic potential within specific microbial consortia, taking into account the inherent differences within and among different ethnic groups [160], sexual and hygiene practices, and host susceptibility [23]. It has been proposed that more information regarding the aetiology of BV may be attained by aggregating the microflora into higher taxonomic groups instead of considering the vaginal microflora at genus or species level [151] and that if the occurrence of BV is reduced and a healthy vaginal biofilm maintained, new cases of HIV infections may be considerably prevented [161] as will adverse pregnancy outcomes. Studies demonstrating the ability of specific bacteria to overcome immunity and alter the environment (be it synergistically or antagonistically) in favour of their survival, will greatly improve our understanding of the complexity of BV [39,47] and the microbial stimuli of inflammatory reactions which may initiate the cascade of events leading to preterm delivery. It is therefore imperative that a better understanding of the vaginal microbiome with a more explicit differentiation between health and disease microbiota be established, taking into account other risk factors which may modify their colonisation and pathogenic potential.
